# Cannabis Mobile Apps: A Content Analysis

**DOI:** 10.2196/mhealth.4405

**Published:** 2015-08-12

**Authors:** Danielle E Ramo, Lucy Popova, Rachel Grana, Shirley Zhao, Kathryn Chavez

**Affiliations:** ^1^ Department of Psychiatry University of California, San Francisco San Francisco, CA United States; ^2^ Center for Tobacco Control Research and Education University of California, San Francisco San Francisco, CA United States

**Keywords:** cell phones, mobile apps, cannabis

## Abstract

**Background:**

Mobile technology is pervasive and widely used to obtain information about drugs such as cannabis, especially in a climate of rapidly changing cannabis policy; yet the content of available cannabis apps is largely unknown. Understanding the resources available to those searching for cannabis apps will clarify how this technology is being used to reflect and influence cannabis use behavior.

**Objective:**

We investigated the content of 59 cannabis-related mobile apps for Apple and Android devices as of November 26, 2014.

**Methods:**

The Apple and Google Play app stores were searched using the terms “cannabis” and “marijuana.” Three trained coders classified the top 20 apps for each term and each store, using a coding guide. Apps were examined for the presence of 20 content codes derived by the researchers.

**Results:**

Total apps available for each search term were 124 for cannabis and 218 for marijuana in the Apple App Store, and 250 each for cannabis and marijuana on Google Play. The top 20 apps in each category in each store were coded for 59 independent apps (30 Apple, 29 Google Play). The three most common content areas were cannabis strain classification (33.9%), facts about cannabis (20.3%), and games (20.3%). In the Apple App Store, most apps were free (77%), all were rated “17+” years, and the average user rating was 3.9/5 stars. The most popular apps provided cannabis strain classifications (50%), dispensary information (27%), or general facts about cannabis (27%). Only one app (3%) provided information or resources related to cannabis abuse, addiction, or treatment. On Google Play, most apps were free (93%), rated “high maturity” (79%), and the average user rating was 4.1/5. The most popular app types offered games (28%), phone utilities (eg, wallpaper, clock; 21%) and cannabis food recipes (21%); no apps addressed abuse, addiction, or treatment.

**Conclusions:**

Cannabis apps are generally free and highly rated. Apps were most often informational (facts, strain classification), or recreational (games), likely reflecting and influencing the growing acceptance of cannabis for medical and recreational purposes. Apps addressing addiction or cessation were underrepresented in the most popular cannabis mobile apps. Differences among apps for Apple and Android platforms likely reflect differences in the population of users, developer choice, and platform regulations.

## Introduction

Cannabis is the most widely used illicit substance in the United States, with 19.8 million US residents (7.5%) age 12 or older reporting past-month use in 2013 [1]. In 2012, the prevalence of cannabis use surpassed that of cigarette smoking among youth age 12 to 17, and this continued into 2013 and 2014 [1,2]. Cannabis use and its legalization are contested issues, as policy changes have led to increases in the availability of cannabis for medical and recreational use in the United States, and problems associated with using cannabis (eg, diagnoses of cannabis use disorder) [3]. Cannabis remains a Schedule I substance under US federal law; however, two US states have legalized retail cannabis, two additional states and the District of Columbia have passed legislation to legalize use, and 33 states and Guam have legalized medical cannabis use. These state-level policy changes in the United States and the continued tension between federal and state laws have led to a proliferation of cannabis-related information in the United States and across the world.

Mobile technology, including the mobile phone, has been a vehicle for cannabis-related news and information. With a global audience totaling up to 1.75 billion in 2014, mobile phone technology is pervasive and widely used [4]. Mobile phones have revolutionized mobile communication technology through the availability of Internet access. Mobile phones also allow users to download apps, which are programs designed specifically for mobile phone operating systems. In June 2014, Apple announced that 75 billion apps had been downloaded from its App Store for the iPhone/iPad [5], and a June 2014 report showed that downloads from Google Play for Android devices had reached roughly 80 billion [6]. Market research firms have estimated that in 2015, there will be nearly 3 billion devices running the Android operating system and 500 million running the Apple operating system (iOS) worldwide [6].

People are increasingly turning to mobile phones to get information about potential health risk behaviors such as cannabis use. A majority of users (52% of Americans) use their mobile phones to gather health-related information [7]. However, it is unclear what is available to them when they do seek out this information.

Some previous work has characterized the content of mobile apps related to substance use. A review of 384 alcohol-related apps available on Apple’s App Store and Google Play found that the majority of apps were primarily for entertainment (50%) or claimed to provide a blood alcohol concentration (39%), and the latter apps were highly unreliable. Only 11% of apps supported the safety of reduction/cessation of drinking [8]. A review of 87 addiction apps on Google Play in 2012 found that apps typically provided information on recovery, content to enhance motivation and promote social support, and tools to monitor progress [9]. Abroms and colleagues conducted two reviews of mobile apps for tobacco cessation, and concluded that these apps generally do not adhere to US Clinical Practice Guidelines for smoking cessation [10,11]. Jacobs and colleagues [12] classified Facebook apps for smoking cessation, similarly concluding that the few available apps had low adherence to recommended guidelines. There has not been a content analysis of mobile apps related to cannabis use, and it is unclear whether available apps address addiction or cessation at all.

Other content analyses have evaluated the scientific rigor of health-related mobile apps. Cowan et al [13] found that iPhone apps targeting physical activity were generally lacking in theoretical content, and higher-priced apps and those that addressed a broader activity spectrum incorporated more theoretical content. In an evaluation of iPhone diet apps, West et al [14] found most apps to be theory deficient and provide only general information or assistance. Breton and colleagues similarly concluded that only a small fraction (15%) of apps for weight control adhered to 5 or more of 13 practices recommended by government agencies for the control of weight [15]. Content analysis of apps for pediatric obesity prevention (weight loss, healthy eating, physical activity) found that most apps (62%) lacked any expert recommendations, and those that did were limited in the number of recommendations made (mean 3.6, SD 2.7 out of 15 defined guidelines). More than half (56%) of the apps were games, consistent with strategies appealing to young people [16]. Other reviews showed a similar lack of comprehensive, scientific information for asthma management [17], and variance in adherence to established guidelines for first aid by source [18] and for cancer information by target audience (health care professionals vs the public) [19]. Results of these studies highlight that, although the material is vast, there is a lack of information with scientific grounding or theory-based interventions available to users.

A report of young adults’ perspectives on apps for health behavior change showed that young adults have an interest in using such apps, and that accuracy, legitimacy, security, effort required, and immediate effects on mood were important influences on app usage [20]. It is unclear whether mobile apps for cannabis use address health behavior change in any way. Given widespread cannabis use, political controversy surrounding its legalization, and the potential for mobile technology to deliver information about cannabis to a large body of users worldwide, there is a need to understand the nature of available cannabis information on mobile phones. This study analyzed the top cannabis-related mobile apps on Apple’s App Store and Google Play in terms of app characteristics (price, rating, download range) and content codes (strain classification, laws, games, social media, medicinal use, etc). Findings will inform how cannabis is portrayed in the context of mobile apps, describe what (if any) health information is conveyed in cannabis apps, and highlight gaps in available information.

## Methods

### Identifying Cannabis Apps

We used several strategies to identify apps that would be most likely encountered by users seeking cannabis-related information. First, a search for the most commonly used words for “cannabis” was performed at the website UrbanDictionary.com in January 2014. A total of 110 commonly used and slang terms were listed, and used as search terms on Apple’s App Store and Google Play. At the time of searching, Google Play reported up to 250 apps for each term and all terms had 250 results. On Apple’s App Store, the number of results differed widely by search term. Terms with meanings other than strictly cannabis (eg, “bomb,” “zombie,” “pot”) had more results than those referring primarily to cannabis (eg, “marijuana”). The first, second, and third authors made a decision not to use terms that could refer to something other than cannabis, and used the frequency of search results for all remaining terms to inform the final choice to use the terms “cannabis” and “marijuana” for the content analysis.

A search for apps using the keywords “marijuana” and “cannabis” was then performed on Apple’s App Store and Google Play on November 26, 2014. The first 20 results for either search term in both stores were considered for coding. Any app that did not duplicate a previous result was coded for content. Only the first 20 results were chosen for analysis based on consensus among investigators that users rarely go beyond the first 1-2 screens in app stores, a rationale similar to that used in a content analysis of electronic cigarette websites [21]. The total number of apps analyzed in this exploratory project was comparable to previous reviews of tobacco smoking cessation apps that included 47 [10] and 98 apps [11], and a review of 12 Facebook apps for smoking cessation [22].

### Coding Guide Development

In January 2014, a research staff member, supervised by the first author, searched the Apple App Store and Google Play store using the term “marijuana” and selected the top 10 apps in each store (20 apps total) to develop a coding guide, similar to the procedure used by Grana and Ling [21] for electronic cigarette websites. The staff first drafted a guide relevant to cannabis apps, including prompting for all features included on the Apple App Store and Google Play, and content codes. In March 2014, a coder who did not participate in the final coding process for the analysis again selected a sample of top 10 apps from each store using the search term “marijuana” (for a total of 20 apps), coded them and further revised the coding guide. The top apps listed for searches change quickly; therefore, the apps that were in the top search results during the coding guide development may not have been in the top results at the time of the final content analysis.

Throughout the coding guide development process, the guide was reviewed iteratively by the first three authors, refined, and retested to generate consistent definitions and examples.

In March 2014, 2 coders were trained by the first author and a sample of apps were coded and evaluated for reliability. Twenty apps (10 Apple’s App Store, 10 Google Play, not necessarily included in the final sample) resulting from the search term “marijuana” were used for training, and reliability with Kappa ranged from .62 (medicinal use) to .89 (news). Any discrepancies in coding were discussed with the first author and a consensus was reached. After reliability was established, apps meeting search criteria for the main analysis (search terms “cannabis” and “marijuana”) were divided and given to the same 2 coders. Coders downloaded all free and paid apps to examine features. In November 2014, a third coder downloaded and coded all 59 apps reported in this analysis. Reliability for the 8 apps that were the same on Apple’s App Store under the search term “cannabis” in March and November 2014 ranged from .71 (facts) to 1.00 (all other categories), indicating strong reliability.

### Coding Guide

The final guide included coding for basic information about app compatibility (eg, type of mobile phone, version of operating system required for use), and basic description of the app based on information reported by Apple’s App Store or Google Play (name of the app, URL, age restriction based on Apple’s App Store or Google Play categories, average user ratings based on Apple’s App Store or Google Play ratings, total app installs [Google Play only], and any exact fees for app use). Categories of apps as specified by Apple’s App Store or Google Play were also coded. Explanations of Apple App Store categories were available online [23]. No description of categories was available for Google Play.

Each mobile app was coded for the presence of each of the following content codes: (1) *Utilities* (including phone wallpaper, battery widget, backgrounds, clock widget, weather widget, brightness widget, toggle widgets [wifi, sound, auto-rotate, data], unit converter weight scale); (2) *News* (cannabis-related); (3) *Social Media* (apps allowing for connection with other users); (4) *Medicinal Use* (connection with doctors who prescribe medicinal cannabis); (5) *Recipes* for cooking with cannabis; (6) *Games* (cannabis-themed); (7) Cannabis *Strain Classification* (pictures, videos, information about the effects of each strain, ability to upload pictures of cannabis); (8) Information on *Growing Cannabis* (eg, information on seed fermentation or ideal growing conditions); (9) *Dispensaries* (medical or recreational, global vs specific region, contacting a dispensary, offer of discounts for dispensary products); (10) *Laws* pertaining to cannabis (including in any US state or another geographic region); (11) Cannabis *Social Gatherings* (descriptions, directions to cannabis events); (12) *Cannabis Cup* results; (13) Cannabis *Smoking Etiquette* (eg, “how” to smoke, things not to do); (14) Cannabis *Dictionary*; (15) *Facts* about cannabis; (16) Cannabis *Abuse, Addiction or Treatment*; (17) *Virtual Simulation* (eg, smoking a virtual joint, realistic joint rolling); (18) *Log* of cannabis use (record of previously tried marijuana/cannabis strains and/or flavors, daily use of joints/blunts, and/or daily growth of marijuana/cannabis plants); (19) Cannabis *Jokes*; and (20) Cannabis Q*uotes*. A single app could include any number of categories. Examples of each content code are listed in Table 2, and screenshots of sample apps are in Multimedia Appendix 1.

### Data Analysis

We calculated the frequency of results from each search term related to cannabis and determined the best search terms for content coding. Once coding was completed, we calculated frequencies and used descriptive statistics to characterize apps. Microsoft Excel was used for all analyses.

## Results

### Content Analysis

Total apps available for each search term were 124 for “cannabis” and 218 for “marijuana” on Apple’s App Store, and 250 each for “cannabis” and “marijuana” on Google Play. Within the top 20 apps for each search term (“cannabis” and “marijuana”) in each store, there were 10 duplicates on Apple’s App Store and 11 duplicates on Google Play. Thus, 59 independent apps (30 on Apple’s App Store, 29 on Google Play) were coded (see Appendix 2 for a complete list).

On Apple’s App Store, most apps were free (77%), all were rated for users aged “17+” years, and the average rating was 3.9/5 stars. On Google Play, most apps were free (93%), rated “high maturity” (79%), and the average rating was 4.1/5 stars. The modal range of downloads was 100,000-500,000 (34%; Table 1).

**Table 1:** Overview of mobile cannabis/marijuana apps (N=59).

Category		All (N=59)	Apple App Store (n=30)	Google Play (n=29)
Free, n (%)		50 (85)	23 (77)	27 (93)
Average price^a^ (SD)		$2.43 (0.88)	$2.56 (0.79)	$1.99 (1.41)
Average user rating (SD)		4.0 stars (0.64)	3.9 stars (0.94)	4.1 stars (0.31)
**Type of app ^b^, n (%)**				
	Lifestyle	17 (29)	12 (40)	5 (17)
	Medical	11 (19)	9 (30)	2 (7)
	News (App Store)/ news & magazines (Google Play)	3 (5)	2 (7)	1 (3)
	Games	11 (19)	4 (13)	7 (24)
	Reference (App Store)/books & reference (Google Play)	5 (9)	2 (7)	3 (10)
	Health & fitness	3 (5)	1 (3)	2 (7)
	Personalization^c^	N/A	N/A	6 (21)
	Education^c^	N/A	N/A	1 (3)
	Entertainment^c^	N/A	N/A	2 (7)
**Age restriction/maturity level, n (%)**				
	17+	N/A	30 (100)	N/A
	High maturity	N/A	N/A	23 (79)
	Medium maturity	N/A	N/A	6 (21)
**Total installs ^d^, n (%)**				
	1000-5000	N/A	N/A	2 (7)
	5000-10,000	N/A	N/A	2 (7)
	10,000-50,000	N/A	N/A	6 (21)
	50,000-100,000	N/A	N/A	2 (7)
	100,000-500,000	N/A	N/A	10 (34)
	500,000-1,000,000	N/A	N/A	5 (17)
	1,000,000-5,000,000	N/A	N/A	2 (7)

^a^Average price calculated for only those apps with any cost.

^b^Type of app as specified by Apple’s App Store or Google Play.

^c^Category only present on Google Play.

^d^ Installs information only present on Google Play.

Based on our coding, the three most common content codes across all apps were cannabis strain classification (33.9%), facts about cannabis (20.3%), and games (20.3%). On Apple’s App Store, the top 20 apps provided cannabis strain classifications (50%), dispensary information (27%), or general facts about cannabis (27%; Table 2). Only one app (3%) provided any information or resources related to cannabis abuse, addiction, or treatment. On Google Play, the most popular apps offered games (28%), phone utilities (eg, wallpaper, clock; 21%) and cannabis food recipes (21%); no apps addressed abuse, addiction, or treatment.

**Table 2:** Content codes of mobile cannabis apps (N=59).

	Example	All, n (%) (N=59)	Apple App Store, n (%) (n=30)	Google Play, n (%) (n=29)
Strain classification	*Leafly Marijuana Strain and Dispensary Reviews* (App Store and Google Play)—contains a database of hundreds of marijuana strains and their effects, flavors, medical treatment, and availability nearby	20 (34)	15 (50)	5 (17)
Facts	*Marijuana Facts* (Google Play)—a collection of marijuana-related facts pertaining to consumption, cultivation, production, history, and sustainability	12 (20)	8 (27)	4 (13)
Game	*Pot Farm - Grass Roots* (Google Play)—grow and sell virtual weed	12 (20)	4 (13)	8 (28)
Dispensaries	*Weedmaps* (App Store and Google Play)—helps users connect to local dispensaries and access menus, strain reviews, and exclusive offers	10 (17)	8 (27)	2 (7)
Recipes	*Weed Cookbook - Medical Marijuana Recipes & Cooking* (App Store)—a collection of recipes that include cannabis as an ingredient	10 (17)	4 (13)	6 (21)
News	*Cannabis News Pro* (App Store)—a collection of cannabis-related news from multiple sources	9 (15)	6 (20)	3 (10)
Utilities	*Cannabis Joint Battery Widget* (Google Play)—shows phone’s battery level as a burning joint	9 (15)	3 (10)	6 (21)
Growing cannabis	*Cannabis Pocket Reference* (Google Play)—contains a Grow Guide with growing information, pest/disease control tips, grow diary, and nutrient charts	8 (14)	4 (13)	4 (14)
Social media	*Rate My Weed - The First Ever Marijuana Recognition Software* (App Store)—allows users to share marijuana recognition results on Facebook and Twitter	8 (14)	6 (20)	2 (7)
Laws	*WeedLaws: Marijuana Law Guide* (Google Play)—provides state-specific legal information regarding use, possession, cultivation, and sale of cannabis	7 (12)	4 (13)	3 (10)
Medicinal use	*Marijuana - MyGreenz Locator* (App Store)—connects users to medical marijuana doctors and dispensaries	7 (12)	5 (17)	2 (7)
Cannabis cup	*Cannabis Cups* (App Store)—allows users to view and judge featured strains, gives directions to Cannabis Cup events, and includes Cannabis Cup results from previous years	5 (9)	4 (13)	1 (3)
Log	*Medical Marijuana Log* (App Store)—allows users to record daily cannabis use via blunts, joints, vapes, bowls, or dabs	5 (9)	4 (13)	1 (3)
Dictionary	*Marijuana 420* (App Store)—dictionary of marijuana-related terms, street names, and slang	3 (5)	3 (10)	0
Quotes	*Marijuana Quotes* (Google Play)—collection of marijuana-related quotes	3 (5)	0	3 (10)
Smoking etiquette	*Joint 4 Dummies* (Google Play)—teaches users several ways to roll a joint	3 (5)	2 (7)	1 (3)
Social gatherings	*Cannabis Culture* (Google Play)—Canadian-based magazine that covers cannabis cultural events such as the Global Marijuana March and 420	3 (5)	2 (7)	1 (3)
Virtual simulation	*iSmoke: Weed HD - Free* (Google Play)—a simulation of rolling and lighting a joint while listening to a soundtrack	2 (3)	0	2 (7)
Abuse, addiction, or treatment	*Marijuana Anonymous Mobile* (App Store)—12-step guide to recovering from marijuana addiction, includes addiction-related literature and a meeting search function to connect users	1 (2)	1 (3)	0
Jokes	*Marijuana Jokes* (Google Play)—collection of marijuana-related jokes	1 (2)	0	1 (3)

On Apple’s App Store, the app that appeared as the top result for both search terms (“cannabis” and “marijuana”) was *Leafly Marijuana Strain and Dispensary Reviews*. It featured a large database of strain reviews (with pictures, flavors, effects, etc), a “Finder” function to locate and review nearby dispensaries (medical or recreational), and an information section with cannabis-related news and content. The app was free and had an average user rating of 5 stars. This app was given 5 content codes: *News*, *Medicinal Use*, *Strain Classification*, *Dispensaries*, and *Facts*.

The app that appeared as the second result for both search terms in Apple’s App Store was *Marijuana Handbook Lite - The Ultimate Medical Cannabis Guide With The Best of Edible, Ganja Strains, Weed Facts, Bud Slang and More!* The app featured a wide range of cannabis-related resources, including a strain library, a maps section to locate medical or recreational dispensaries, a marijuana dictionary, a facts section, and a cookbook. The app was free and the average user rating was unknown. This app was given 6 content codes: *Recipes*, *Strain Classification*, *Dispensaries*, *Smoking Etiquette*, *Dictionary*, and *Facts*.

On Google Play, the app that appeared as the top result for both search terms was *Pot Farm - Grass Roots*, a game that allowed players to grow and harvest virtual marijuana. Users could run their own dispensaries to sell weed and connect with other gamers to trade weed. The app was free, had an average user rating of 4.4 stars, and had 500,000-1,000,000 downloads. This app was given 2 content codes: *Social Media* and *Games*.

The Google Play app that appeared as the second result for the search term “cannabis” was *BudTrimmer - Weed and Cannabis*, a game in which a player swiped across the screen to slash buds and collect points. The app was free, had an average user rating of 4.3 stars, and had 100,000-500,000 downloads. This app was coded as a *Game*.

The Google Play app that appeared as the second result for the search term “marijuana” was *Weedmaps*. This app functioned as a dispensary finder that helped users connect to local medical or recreational dispensaries, medical marijuana doctors, and delivery services. Users could also access dispensary menus, strain reviews, and “exclusive offers.” The app was free, had an average user rating of 4.2 stars, and had 1,000,000-5,000,000 downloads. Coders gave this app 4 content codes: *Medicinal Use*, *Strain Classification*, *Dispensaries*, and *Facts*.

Only one app addressed cannabis abuse, addiction, or treatment in any way. On Apple’s App store, *Marijuana Anonymous Mobile* employed a 12-step recovery program, analogous to the 12-Step Recovery Program of Alcoholics Anonymous, to help users stop cannabis use. The app contained information regarding addiction and recovery, including a monthly newsletter and an interactive 12-Step Workbook. It offered a chat feature and a meeting search function that allowed users to find in-person, phone, and online meetings. It also included a Sobriety Date Counter that allowed users to record the number of days of abstinence. The app was listed as the 17^th^ result under the search term “marijuana,” was free, and had an unknown user rating. This app was given 2 content codes: *Abuse, Addiction, or Treatment* and *Facts*.

## Discussion

### Principal Findings

This study examined 59 mobile apps related to cannabis. Over half (58%) of the top apps on Google Play had been downloaded at least 100,000 times, showing that mobile users are generally interested in this category of apps. Overall, the most popular mobile app content was primarily focused on information (strain classification, facts) and recreation (games), with cannabis recipes demonstrating popularity as well. In contrast, only one app in our sample focused on cannabis addiction or treatment. The popularity of informational and recreational apps likely reflects a growing societal acceptance of cannabis, which is also reflected in state laws that have become more favorable toward medical and recreational use in recent years. Indeed, Apple recently relaxed a policy in its App Store prohibiting cannabis-related social media apps, likely both reflective of and influencing the increasing acceptance of cannabis use as something that connects a growing number of people [24]. To our knowledge, this study is the first to describe the content of cannabis-related mobile apps. Updates are likely to show a larger number of apps and a continued focus on information and recreation if state laws and public attitudes toward cannabis continue to move in a favorable direction.

Apps addressing addiction or cannabis cessation were underrepresented in the most popular cannabis mobile apps. This could in part reflect the low level of motivation among non-treatment-seeking marijuana users to cut down or quit use [25]. It also could reflect a lack of interest among mobile phone users to seek out health-related or cessation apps pertaining to cannabis. Although there is some evidence that a majority (57%) of clients in drug treatment have mobile phones [26], there is limited evidence about the desire of persons in treatment to use apps for therapeutic purposes and about the effectiveness of mobile apps for cannabis treatment. We found two published studies that showed promise in the use of mobile technology to treat cannabis use disorders: one using an ecological momentary intervention to monitor substance use among youth after a treatment episode [27]; and another testing the usability of an app to monitor and reduce cannabis use (Assess Plan Track Tips) [28]. As apps are developed, if evidence mounts as to their success in aiding behavior change, it is possible that these apps could become a reliable source of cannabis education or cessation interventions. Health departments and other prominent health organizations should consider creating or endorsing accurate and evidence-based cannabis mobile apps to give them credibility in the ever-expanding app marketplace.

The top apps for iPhone tend to provide information (eg, strains of cannabis) or have some educational purpose, while top Android apps tend to be primarily for entertainment (games, phone utilities). These differences likely reflect differences between iPhone and Android developers, who often program an app for only one platform, and the platforms themselves dictate app content based on rules/approval. For example, the Android market is perceived to be easier to enter than the Apple market because of fewer restrictions [6]; scrutiny may be particularly strong for approving cannabis apps in the Apple App Store [29].

Although we could not link user data to app characteristics in this analysis, market research has suggested that while Android has a larger user base, iPhone users in the United States are younger and more affluent, engage with a larger amount of mobile phone content, and are more likely to engage in mobile commerce [30]. Future investigations should directly survey mobile users about their use of cannabis apps on both Apple and Android operating systems to determine potential implications for user activity. Of the 59 apps coded for this project, 8 were available on both platforms (Apple and Android). Of those 8, 6 were in the top 20 apps under either “marijuana” or “cannabis” search terms on the Apple App Store, but not in the top 20 on Google Play. *Leafly Marijuana Strain and Dispensary Reviews* and *Weedmaps* were in the top 20 in both stores using all 4 search terms, indicating popularity among users of both types of devices. Both apps provide a resource for those looking to classify different types of cannabis and locate dispensaries.

### Limitations

It was not possible to link app content to user data. Further, search criteria were generated by using common words related to cannabis (ie, “cannabis” and “marijuana”); however, users who are seeking a particular type of app may search directly for that app type (eg, “cannabis treatment”), which would not have been captured by this analysis and would likely yield different results. Surveys of cannabis app seekers would be useful to clarify this further. The decision to rate the top 20 app results for each search term was based on the authors’ judgment that this would encompass apps seen by a user during most searches. The logic was that most users would not go beyond approximately 1-2 screens on a desktop computer and even fewer results on a mobile device, for which fewer results are available on a single screen. A different method, incorporating more apps, would likely have yielded different results. The content analysis is based on search results found in November 2014. The app marketplace is changing rapidly; therefore, apps that appeared at the top of search at the time of the study may no longer be top apps in the future.

### Conclusions

Cannabis mobile apps are numerous and the most popular apps focus on information, classification, and recreation (eg, games), consistent with the expanding business of growing and selling cannabis in the United States. Notably absent from the most popular apps were those addressing the important public health concern of cannabis addiction or negative health effects, for which mobile apps could be useful. Finally, there are notable differences among apps on Apple’s App Store and Google Play, likely reflecting the population of users, developer choice, and platform regulations.

## Appendix

Multimedia Appendix 1 Screenshots of apps representing each content area coded.

Multimedia Appendix 2 List of mobile apps analyzed (N=59) in order of popularity.
