# Supplementary material for: Cannabis Mobile Apps: A Content Analysis
Source: JMIR Mhealth Uhealth. 2015 Aug 12;3(3):e81. doi: 10.2196/mhealth.4405 (PMC4705020; doi:10.2196/mhealth.4405)
Supplement: Multimedia Appendix 1 [file mhealth_v3i3e81_app1.pdf]

Appendix 1: Screenshots of apps representing the 11 most popular content areas coded, in order of popularity

Strain Classification

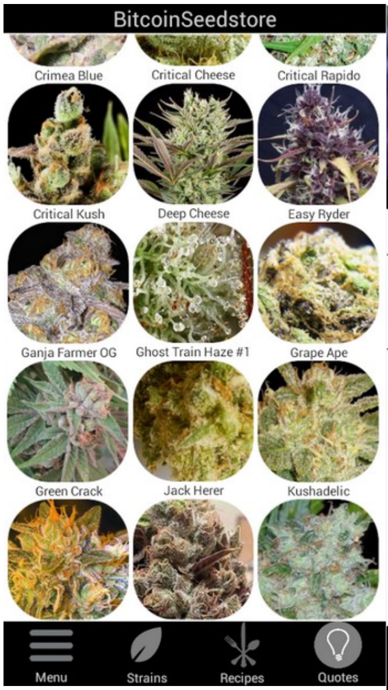


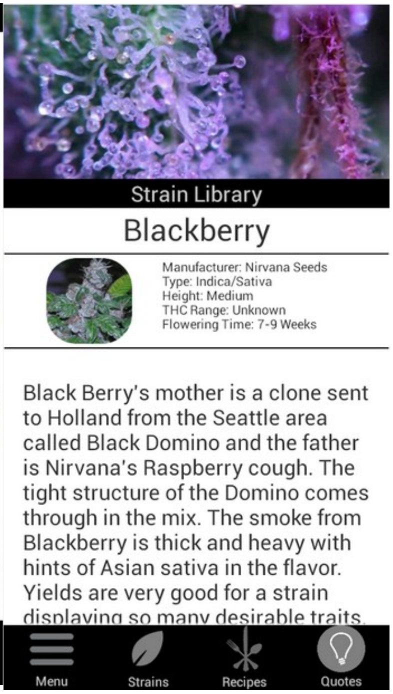


Marijuana Strain Guide (Google Play)
https://play.google.com/store/apps/details?id=com.mobincube.android.sc_9Y7S3

Facts

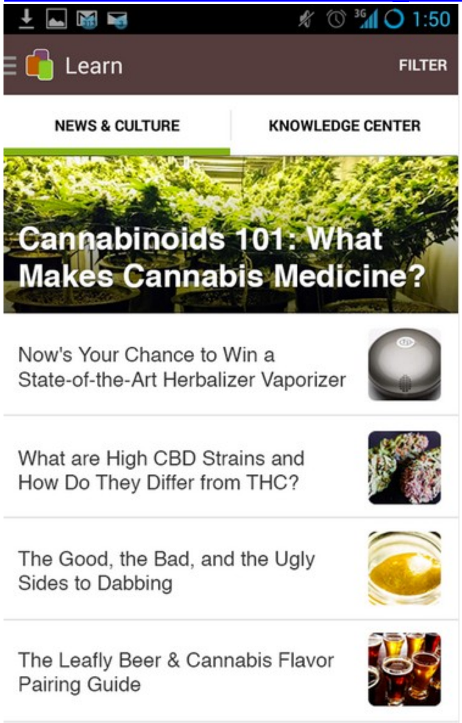


Leafly Marijuana Reviews (Google Play)
https://play.google.com/store/apps/details?id=leafly.android

Games

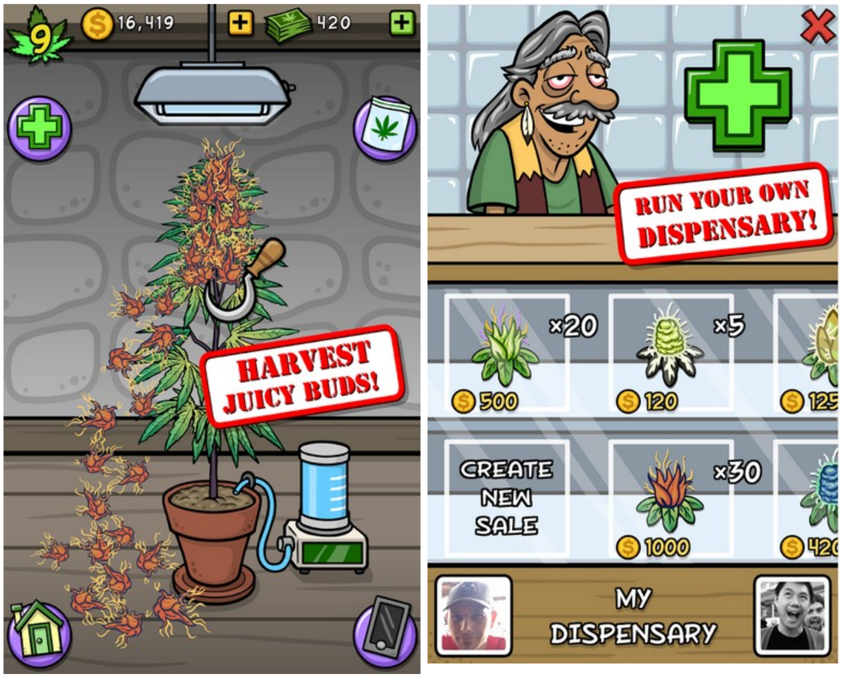


Pot Farm – Grass Roots (Google Play)
[https://play.google.com/store/apps/details?id=air.com.eastsidegamestudio.PFGrassRoots](https://play.google.com/store/apps/details?id=air.com.eastsidegamestudio.PFGrassRoots)

Dispensaries

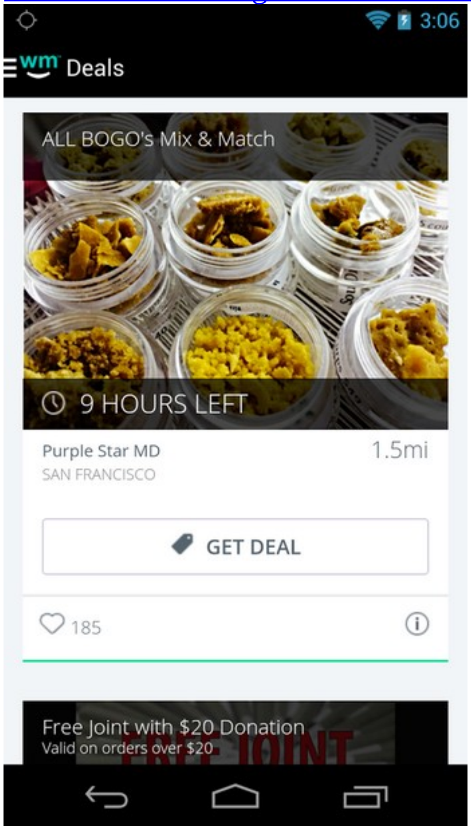


Weedmaps (Google Play)
https://play.google.com/store/apps/details?

id=com.weedmaps.app.android

Recipes

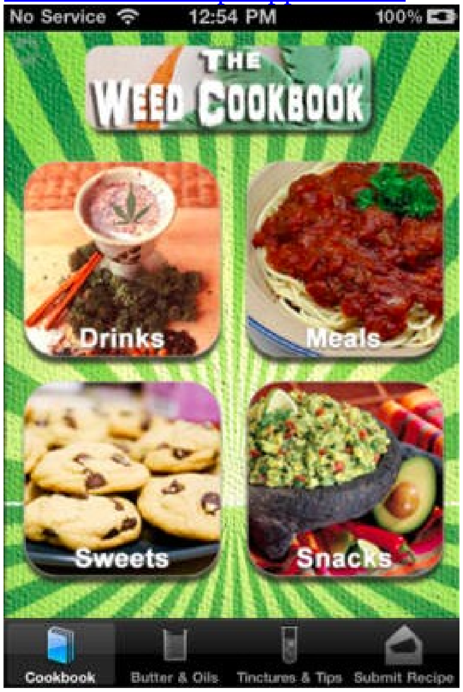


Weed Cookbook – Medical Marijuana Recipes & Cooking (Apple's App Store)
https://itunes.apple.com/us/app/weed-cookbook-medical-marijuana/id436743274?mt=8

News

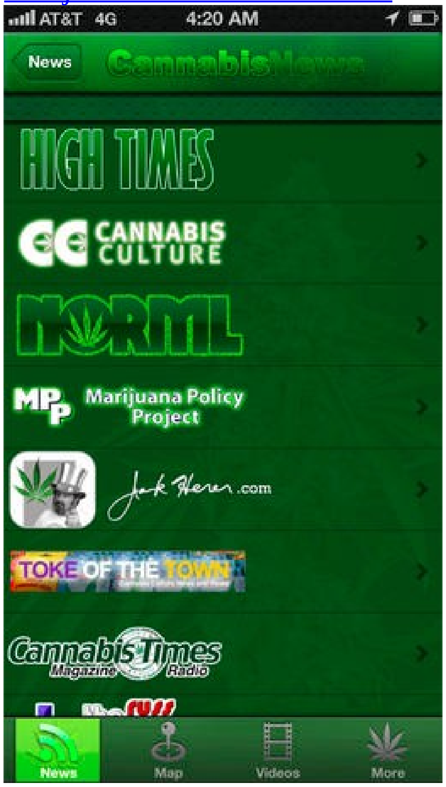


Cannabis News Pro (Apple's App Store)
https://itunes.apple.com/us/app/cannabis-news-pro/id349292282?

mt=8

Utilities

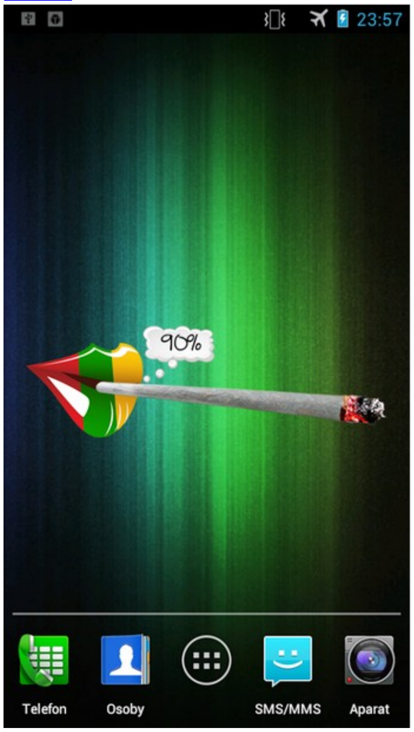


Cannabis Joint Battery Widget (Google Play)
https://play.google.com/store/apps/details?id=org.gp.project.joint.battery

Growing Cannabis

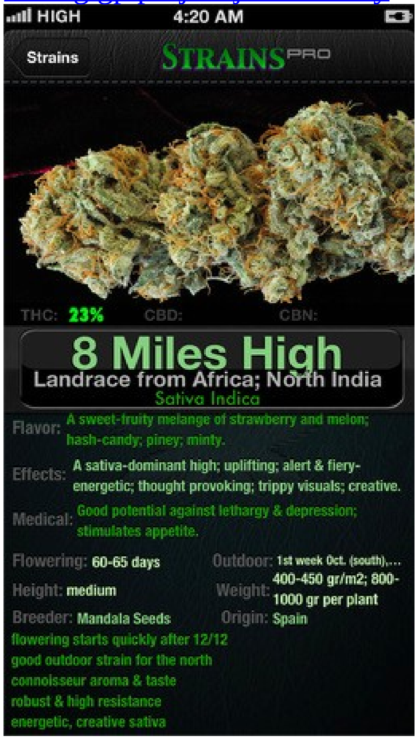


Strains Pro – An Advanced Breeder's Guide to World's Distinctive Cannabis (Apple's App Store)
https://itunes.apple.com/us/app/strains-pro-advanced-

[breeders/id383647497?mt=8](breeders/id383647497?mt=8)

Social Media

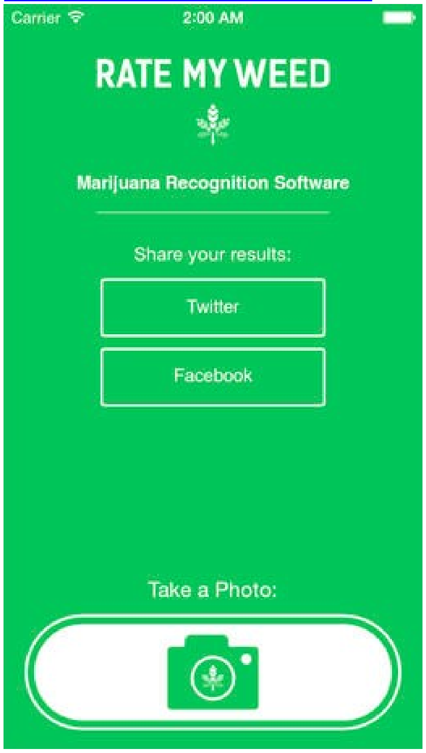


Rate My Weed – The First Ever Marijuana Recognition Software (Apple's App Store)
[https://itunes.apple.com/us/app/rate-my-weed-first-ever-marijuana/id842374308?mt=8](https://itunes.apple.com/us/app/rate-my-weed-first-ever-marijuana/id842374308?mt=8)

Laws

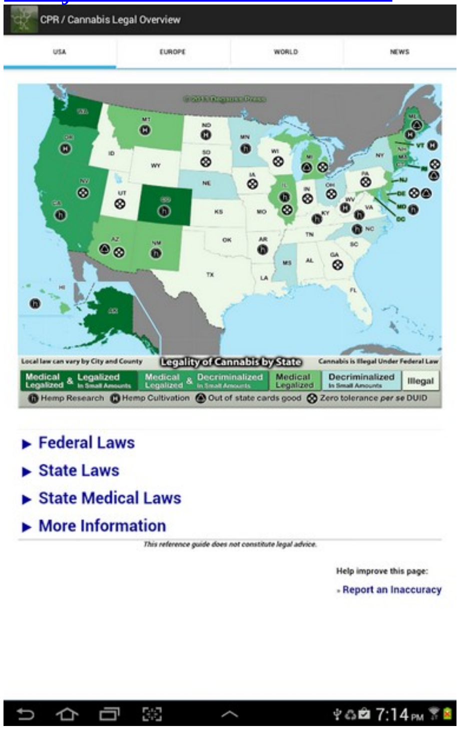


Cannabis Pocket Reference (Google Play)
[https://play.google.com/store/apps/details?](https://play.google.com/store/apps/details?)

id=com.degausspress.CPR

Medicinal Use

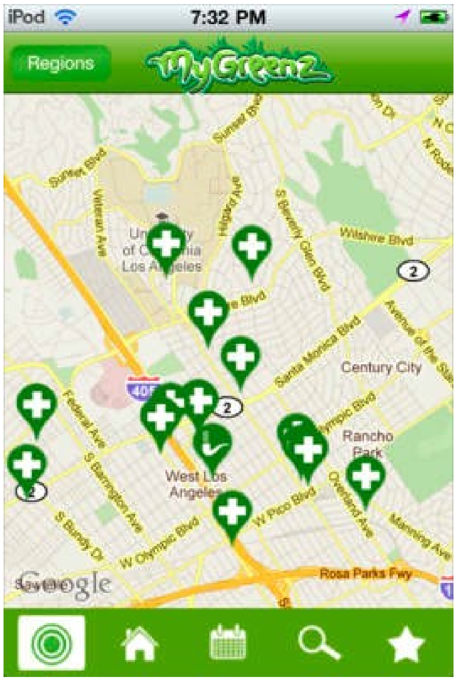


Marijuana – MyGreenz Locator (Apple’s App Store)
https://itunes.apple.com/us/app/marijuana-mygreenz-locator/id443222909?mt=8
