# Supplementary material for: Cannabis Mobile Apps: A Content Analysis
Source: JMIR Mhealth Uhealth. 2015 Aug 12;3(3):e81. doi: 10.2196/mhealth.4405 (PMC4705020; doi:10.2196/mhealth.4405)
Supplement: Multimedia Appendix 2 [file mhealth_v3i3e81_app2.pdf]

Appendix 2: List of Smartphone Apps Analyzed (N=59) in order of popularity

Apple's App Store

Name	App Type	URL
Leafly Marijuana Strain and Dispensary Reviews	Medical	https://itunes.apple.com/us/app/leafly-marijuana-strain-dispensary/id416456429?mt=8
Marijuana Handbook Lite	Lifestyle	https://itunes.apple.com/us/app/marijuana-handbook-lite-ultimate/id477750914?mt=8
Frweed+	Medical	https://itunes.apple.com/us/app/frweed-+/id388027395?mt=8
STRAINS Pro	Medical	https://itunes.apple.com/us/app/strains-pro/id383647497?mt=8
Cannabis News Pro	News	https://itunes.apple.com/us/app/cannabis-news-pro/id349292282?mt=8
Weedmaps	Medical	https://itunes.apple.com/us/app/weedmaps/id350189835?mt=8
Cannabis Now	Lifestyle	https://itunes.apple.com/us/app/cannabis-now/id662121602?mt=8
Frweed Pro	Medical	https://itunes.apple.com/us/app/frweed-pro/id414815985?mt=8
Best Buds	Lifestyle	https://itunes.apple.com/us/app/best-buds-nug-pictures-weed/id783196962?mt=8
Weed Tycoon	Games	https://itunes.apple.com/us/app/weed-tycoon-ultimate-marijuana/id595106035?mt=8
Cannabis Cups	Reference	https://itunes.apple.com/us/app/cannabis-cups/id487217792?mt=8
Plant Life	Lifestyle	https://itunes.apple.com/us/app/plant-life-review-cannabis/id693830962?mt=8
Leafstrain	Lifestyle	https://itunes.apple.com/us/app/leafstrain-cannabis-strains/id906031084?mt=8
Marijuana Handbook	Lifestyle	https://itunes.apple.com/us/app/marijuana-handbook-ultimate/id470296810?mt=8
Ganja Farm	Games	https://itunes.apple.com/us/app/ganja-farm/id834861892?mt=8
Mega Marijuana Cookbook and Medicinal Cannabis Guide	Lifestyle	https://itunes.apple.com/us/app/mega-marijuana-cookbook-medicinal/id499859359?mt=8
Ultimate Weed Trivia	Games	https://itunes.apple.com/us/app/ultimate-weed-trivia/id858384735?mt=8

Strains Pro 2: World's Best Medical Cannabis Seed Genetics of 2013 Pocket Guide	Medical	https://itunes.apple.com/us/app/strains-pro-2/id670729246?mt=8
News for Marijuana Free HD	News	https://itunes.apple.com/us/app/news-for-marijuana-free-hd/id809686770?mt=8
The Cannabis Daily	Lifestyle	https://itunes.apple.com/us/app/the-cannabis-daily/id888731774?mt=8
Marijuana +	Lifestyle	https://itunes.apple.com/us/app/marijuana-+/id412644728?mt=8
Marijuana 420	Reference	https://itunes.apple.com/us/app/marijuana-420/id413126886?mt=8
Marijuana – MyGreenz Locator	Medical	https://itunes.apple.com/us/app/marijuana-mygreenz-locator/id443222909?mt=8
Weed Yo Screen Free	Lifestyle	https://itunes.apple.com/us/app/weed-yo-screen-free/id444080239?mt=8
Weed Cookbook – Medical Marijuana Recipes & Cooking	Lifestyle	https://itunes.apple.com/us/app/weed-cookbook-medical-marijuana/id436743274?mt=8
Marijuana Dispensary Finder – THC Finder	Medical	https://itunes.apple.com/us/app/marijuana-dispensary-finder/id457072453?mt=8
Medical Marijuana Log	Lifestyle	https://itunes.apple.com/us/app/medical-marijuana-log/id667792077?mt=8
Marijuana Anonymous Mobile	Health & Fitness	https://itunes.apple.com/us/app/marijuana-anonymous-mobile/id874705440?mt=8
Rate My Weed – The First Ever Marijuana Recognition Software	Medical	https://itunes.apple.com/us/app/rate-my-weed-first-ever-marijuana/id842374308?mt=8
Weed Casino	Games	https://itunes.apple.com/us/app/weed-casino-best-marijuana/id776072191?mt=8
